# A TD-DFT Study on the Photo-Physicochemical Properties of Chrysophanol from Rheum

**DOI:** 10.3390/ijms10073186

**Published:** 2009-07-13

**Authors:** Xue Zhao, Zebao Zheng, Shuai Feng, Zhiqiang Shi, Dezhan Chen

**Affiliations:** 1 Department of Chemistry & Environment Science, Taishan University, Taian 271021, China; 2 Department of Materials Science and Chemical Engineering, Taishan University, Taian 271000, China; 3 College of Chemistry & Chemical Engineering, Shandong Normal University, Jinan 250014, China

**Keywords:** TD-DFT, chrysophanol, excited states, photosensitization mechanisms

## Abstract

As a naturally occurring anthraquinone pigment, chrysophanol (MHAQ) has attracted considerable attention in recent years owing to its efficient photosensitivity under the solar spectrum. Considering the successful use of time-dependent density functional theory (TD-DFT) in investigating the photo-physicochemical behaviors of dyes and pigments, we performed a study by means of TD-DFT calculations, which provided us with various excited state properties of chrysophanol, including absorption spectrum, lowest triplet excited-state energy, vertical electron affinity and vertical ionization potential. On the basis of the calculated results, the photosensitive mechanisms of chrysophanol were discussed and some deeper insights were gained. First, we indicated that the experimentally observed chrysophanol’s photo-damage to DNA in oxygen-free media is more likely to arise from MHAQ^•+^ rather than from T_1_ state chrysophanol. Second, we revealed that it is the MHAQ^•−^ that is responsible for the O_2_^•−^ generation in solvents. Based on the photosensitive activities, chrysophanol may be potentially used as the photodynamic medicine for clinical therapy of the diseases occurring on the shallow surface and vascular capillary diseases.

## 1. Introduction

Chrysophanol (3-methyl-1,8-dihydroxyanthraquinone, MHAQ) belongs to a family of anthraquinone pigments that naturally exist in rheum, a Chinese herbal medicine growing abundantly in China. Besides their biological activities, these pigments are also well known as photosensitizers [1,2]. As a family of photosensitizers, the anthraquinone derivatives possess several advantages, such as low costs and efficient photosensitivity under the solar spectrum. Therefore, it may be possible to use them as a group of potential photo-activated pesticides. Furthermore, the absorption spectra of anthraquinones in the visible area are spread over 400—500 nm with log*ɛ* = 4.02 (433 nm), 3.85 (443 nm) and 3.04 (433 nm) for chrysophanol, emodin and mixed anthraquinone derivatives extracted from rheum, respectively [3]. In fact, the light with wavelengths from 400 nm to 500 nm can only penetrate into the tissue no more than 1 mm, therefore, it may be a better choice to apply them for the photodynamic therapy of diseases occurring in shallow surfaces, such as some vascular diseases. These kinds of photosensitizers have been generally neglected because their peak absorption was too short to be excited in the phototherapeutic window (600—900 nm). However, two-photon excitation technique provides the possibility of using a red laser at wavelengths of 800–900 nm to excite the photosensitizers with absorption over short wavelengths, such as from 400–500 nm, for photodynamic therapy of solid tumors and accurately and selectively destroy target tissues [4]. Therefore, anthraquinone pigments, especially chrysophanol, have attracted our attention to study their photochemical properties due to their antitumor and antiviral activities.

As known to all, during photosensitization, pigments in a ground state (S_0_) are initially excited to the singlet excited state (S_1_) and then intersystem cross to the triplet excited state (T_1_). Therefore, some experimental effort has been devoted to investigating the S_1_ and T_1_ state properties of chrysophanol to understand its photosensitizing mechanisms [5,6]. Nevertheless, there is little theoretical study devoted to this topic. Considering the successful use of time-dependent density functional theory (TD-DFT) in investigating the molecular excited-state properties [7–9], we have attempted to perform a study by the TD-DFT method, which will provide some deeper insights into the photosensitizing mechanisms and activity of chrysophanol.

## 2. Methods

All molecular structures were fully optimized using the hybrid B3LYP functional method [10], in combination with the 3-21G and 6-31G(d,p) Gaussian basis set. This method has been proven to give satisfactory results while saving computer time [11]. For each optimized structure a frequency analysis at the same level of theory was used to verify that it corresponds to a stationary point in the potential energy surface. All the calculated frequencies were real. As the use of diffuse functions is essential for accurate determination of the energetics, particularly for ion radicals and excited states [7], the vertical electron affinitie (VEA: the difference between the total electronic energy of parent molecule and anion radical) and vertical ionization potential (VIP: the difference between the total electronic energy of parent molecule and cation radical) were calculated by a combined DFT method B3LYP/6-31+G(d,p)//B3LYP/6-31G(d,p), which means that B3LYP/6-31+G(d,p) was employed to perform a singlet-point calculation on the basis of B3LYP/6-31G(d,p) optimized structures. The excited-state properties were calculated with the time-dependent density functional theory (TD-DFT) formalism, using the optimized ground state geometries. TD-DFT in combination with the B3LYP hybrid functional and the 6-31+G (d, p) basis set has previously been shown to provide accurate energies for excited states within 0.2 eV (5kcal/mol) [12]. The effect of the solvent was included through B3LYP/6-31+G(d, p)//B3LYP/6-31G(d, p) single-point calculations using the polarized continuum model (PCM) [13]. Two different solvents were studied in this work, e = 78.39 and e = 4.34 (corresponding to bulk water and ether environment, respectively). All calculations were performed with the Gaussian 03 package of programs [14].

## 3. Results and Discussion

**Figure 1.**
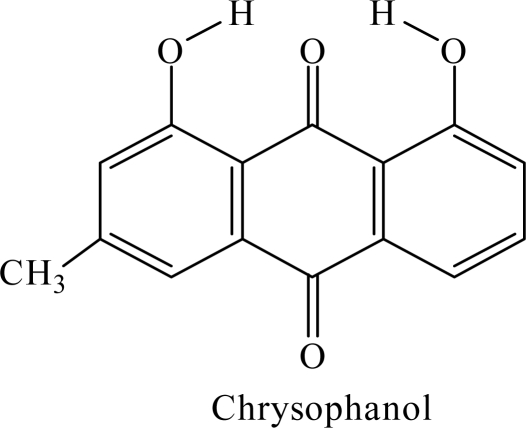
Molecular structure of the chrysophanol (MHAQ).

Chrysophanol (Figure 1) belongs to a family of anthraquinone pigments that naturally exist in rheum. It has gained much attention in recent years owing to its interesting biological and pharmacological activities.

### 3.1. Photo-physicochemical properties of chrysophanol

#### 3.1.1. Singlet excied states

Table 1 list the TD-B3LYP/6-31+G (d, p)-calculated six lowest singlet excitation energies (*E*) and oscillator strengths (*f*) of chrysophanol in vacuum, water and ether. It can be found that there is a strong absorption peak near 429 nm in vacuum. The calculation result is close to the experimental data, which is 433 nm for chrysophanol in gas state [3]. Both in water and ether, we can see the red-shift of absorption band, which occurs especially remarkably in the polar solvent (water).

#### 3.1.2. Triplet excited states

Due to the much longer life time of the T_1_ state than the S_1_ state, the T_1_ state is responsible for the photosensitive reaction. Therefore, the lowest T_1_ excitation energy (*E*_T1_) of a photosensitizer is crucial to understanding its photosensitizing mechanisms. The TD-DFT-calculated *E*_T1_ values of chrysophanol in different medium are listed in Table 2, from which we also find there occurs a certain red-shift of absorption band in solvent comparing with that in vacuum. Interestingly, the *E*_T1_ values are close in ether and water. This suggests that the solvent polarity has little influence on the *E*_T1_ of chrysophanol.

#### 3.1.3. Vertical electron affinities and vertical ionization potentials of chrysophanol

Photosensitizers in the ground and excited states may act as electron-donors or electron-acceptors during photosensitizing reactions. Thus, the vertical electron affinities (VEA) and vertical ionization potentials (VIP) of chrysophanol in S_0_ and T_1_ states have been calculated and are listed in Tables 3 and 4. The VEA in S_0_ state (VEA_S0_) was estimated as −2.73 eV in ether and −2.98 eV in water (Table 3). The differences between them stem from the fact that the anion is better stabilized in polar solvents than in non-polar environments. The VEA in T_1_ state (VEA_T1_ ) for chrysophanol was −4.95 eV in ether and −5.19 eV in water (Table 4), respectively. As shown in Table 3, the VIP in the S_0_ state (VIP_S0_) for chrysophanol was 6.77 eV in ether and 6.53 eV in water, indicating that chrysophanol is more ready to donate electrons in water. In combination with *E*_T1_, VIP in T_1_ state (VIP_T1_ ) for chrysophanol was estimated to be 4.56 eV in ether and 4.32 eV in water (Table 4), respectively. On the basis of these values, the photosensitizing mechanisms of chrysophanol have been investigated.

### 3.2. Elucidation of photosensitizing mechanisms of chrysophanol

It is well known that photosensitization involves two mechanisms, namely, direct reaction with substrates (e.g. DNA, RNA and proteins, mechanism I) or causing damage through oxygen intermediates, via energy transfer or electron transfer to generate toxic ROS (reactive oxygen species, mechanism II).

#### 3.2.1. Mechanism I of chrysophanol photosensitization

Previous studies suggest that chrysophanol can damage DNA upon excitation [4]. In oxygen-free media, mechanism I should be responsible for the photodamage and two possible pathways may be involved. First, T_1_ state chrysophanol can abstract an electron directly from DNA bases [Equation (1)]. Second, chrysophanol radical cation (MHAQ^·+^) can be generated by an autoionization reaction between the T_1_ and S_0_ states [Equation (2)] or both T_1_ states [Equation (3)]. When MHAQ^·+^ is generated, it may accept an electron from DNA [Equation (4)].

(1) $$\text{MHAQ}\;({\text{T}}_{1})+\text{D}\to {\text{MHAQ}}^{•-}+{\text{D}}^{•+}$$

(2) $$\text{MHAQ}\;({\text{T}}_{1})+\text{MHAQ}\;({\text{S}}_{0})\to {\text{MHAQ}}^{•+}+{\text{MHAQ}}^{•-}$$

(3) $$\text{MHAQ}\;({\text{T}}_{1})+\text{MHAQ}\;({\text{T}}_{1})\to {\text{MHAQ}}^{•+}+{\text{MHAQ}}^{•-}$$

(4) $${\text{MHAQ}}^{•+}+\text{D}\to \text{MHAQ}\;({\text{S}}_{0})+{\text{D}}^{•+}$$

Reaction (1) is governed by the VEA_T1_ of chrysophanol and the VIP of bases. If the summation of the two parameters is negative, the reaction is permitted. The VIP of DNA or RNA bases have been calculated by the combined DFT method B3LYP/6-31G(d,p)//B3LYP/6-31G [9], the results are as follows: A, 6.79 eV; G, 6.49 eV; T, 7.36 eV; C, 7.16 eV and U, 7.98 eV in ether and A, 6.18 eV; G, 5.85 eV; T, 6.71 eV; C, 6.60 eV and U, 7.27 eV in water. Thus, the summation of VEA_T1_ of chrysophanol (Table 4 ) and VIP of DNA or RNA bases is positive in ether (>1.54 eV) and water (>0.66 eV), suggesting that the electron transfer between the T_1_ state of chrysophanol and bases is not thermodynamically favorable.

According to the theoretical parameters listed in Table 4, the total energies of reaction (2) (VEA_T1_ + VIP or VIP _T1_ + VEA) for chrysophanol are positive, no matter whether in ether or water. Thus, the MHAQ^•+^ species cannot be generated by reaction (2) and reaction (4) has no chance to occur. However, the total energies of reaction (3) (VEA_T1_ + VIP_T1_) for chrysophanol are negative in both ether and water. So reaction (3) is permitted, owing to the negative reaction energy. When MHAQ^•+^ is formed through reaction (3), it can abstract an electron from adenine and guanine. As the VEA for MHAQ^•+^ is calculated to be −6.52 eV in ether and −6.29 eV in water, once MHAQ^•+^ is formed through reaction (3), it can abstract an electron from G (6.49 eV) in ether and from A or G (6.18, 5.85 eV) in water because of the negative reaction energy. In a word, according to the present calculations, chrysophanol can bring about direct damage to DNA in oxygen-free systems, both in non-polar and polar solvents. The photo-damage to DNA by chrysphanol more likely results from the electron transfer between DNA bases and MHAQ^•+^ rather than from the reaction between bases and T_1_ state chrysphanol. However, as the generation of MHAQ^•+^ depends largely on the concentration of T_1_ state chrysphanol, the DNA damage by MHAQ^•+^ may be trivial compared with that by ROS.

#### 3.2.2. Mechanism II of chrysophanol photosensitization

As to the ROS^–^ associated pathway, first, T_1_ state chrysophanol may react with ground state oxygen (^3^O_2_) through energy transfer to generate singlet excited oxygen (^1^O_2_) [Equation (5)].

(5) $$\text{MHAQ}\;({\text{T}}_{1})+{\;}^{3}{\text{O}}_{2}\to \text{MHAQ}\;({\text{S}}_{0})+{\;}^{1}{\text{O}}_{2}$$

It can be seen from Table 4 that the *E*_T1_ of chrysophanol in water and ether are higher than the excitation energy of ^1^O_2_ (1.06 eV [7]), indicating that the energy transfer between the T_1_ state of chrysophanol and ^3^O_2_ in both solvents are permitted. This agrees well with the fact that chrysophanol can efficiently generate ^1^O_2_ with yields of 0.36 in chloroform (using Φ= 0.84 for HA as a reference.) when irradiated by light [12].

Second, T_1_ state chrysophanol may react with ^3^O_2_ through electron transfer to generate superoxide anion radical (O_2_^•−^) (Equation (6)). The prerequisite of the reaction is that the summation of VIP_T1_ of chrysophanol and the adiabatic electron affinity of ^3^O_2_ (AEA_O2_) is negative.

(6) $$\text{MHAQ}\;({\text{T}}_{1})+{\;}^{3}{\text{O}}_{2}\to {\text{MHAQ}}^{•+}+{\text{O}}_{2}^{•-}$$

However, the summation of VIP_T1_ for chrysophanol (Table 4) and AEA _O2_ (the calculated adiabatic electron affinity of oxygen are −3.91 eV in solution, −0.59 eV in vacuum [7] and −3.14 eV in ether) are positive in all media, suggesting that O_2_^•−^ could not be generated through this pathway in both solvents. Nevertheless, O_2_^•−^ has been indeed observed during the photosensitization of chrysophanol in aqueous buffer [3]. Thus, it is speculated that there exists an alternative O_2_^•−^ generating pathway for chrysophanol, i.e. the electron transfer reaction between MHAQ^•+^ [generated from autoionization, Equation (3)] and ^3^O_2_ [Equation (7)].

(7) $${\text{MHAQ}}^{•-}+{\;}^{3}{\text{O}}_{2}\to \text{MHAQ}\;({\text{S}}_{0})+{\text{O}}_{2}^{•-}$$

In vacuum, reaction (7) is unlikely, because of the positive total reaction energies (AEAO_2_ – VEA_S0_ = 0.84 eV). However, a similar analysis indicates that reaction (7) is permitted in both ether and water owing to the negative reaction energy (−0.41 eV in ether and −0.93 eV in water). Therefore, O_2_^•−^ can be generated by photo-irradiation of chrysophanol in both solvents. Nevertheless, it should be stressed that MHAQ^•−^ is responsible for the O_2_^•−^ generation in solvents. This provides a deeper insight into the O_2_^•−^ generating mechanism of chrysophanol in solvents. Once O_2_^•−^ is available, other ROS, such as H_2_O_2_ and ^•^OH, can be produced through the Fenton reaction [15] or the Haber–Weiss reaction [16], which will efficiently amplify the photosensitizing activity of chrysophanol.

## 4. Conclusions

The photo-physicochemical properties of chrysophanol, including absorption spectrum, lowest triplet excited-state energy, vertical electron affinity and vertical ionization potential, were examined by the TD-DFT method. On the basis of the calculated results, the photosensitive mechanisms of chrysophanol were discussed and some deeper insights were gained. First, we have indicated that the experimentally observed chrysophanol’s photo-damage to DNA in oxygen-free media is more likely to arise from MHAQ^•+^ rather than from T_1_ state chrysophanol. Second, we have revealed that it is the MHAQ^•−^ species that is responsible for the O_2_^•−^ generation in solvents. Based on the photosensitive activities, chrysophanol may be potentially used as the photodynamic medicine for clinical therapy of the diseases occurring on the shallow surface and vascular capillare diseases, while photodynamic mechanism will largely depend on the oxygen content in the target tissue. On the other hand, theoretical methods are useful to investigate the photosensitive behaviors of chrysophanol and can be used to explore photo-physicochemical properties of other anthraquinone pigments.

## Figures and Tables

**Table 1.** Six lowest singlet excitation energies (*E*, ev) and oscillator strengths (*f*) of chrysophanol.

		**S_1_**	**S_2_**	**S_3_**	**S_4_**	**S_5_**	**S_6_**
	***E***	2.8914	2.9121	3.1714	3.4642	3.5143	3.6527
**Vacuum**	**λ**	428.80	424.45	390.95	357.90	352.80	339.43
	***f***	0.2366	0.0000	0.0062	0.0019	0.0000	0.0881
	***E***	2.8235	3.0544	3.1437	3.3537	3.5011	3.6588
**Water**	**λ**	439.12	405.91	394.39	369.70	354.12	338.87
	***f***	0.2902	0.0000	0.0065	0.0063	0.0000	0.1418
	***E***	2.8774	2.9804	3.1681	3.3980	3.5042	3.6550
**Ether**	**λ**	430.88	416.00	391.35	364.87	353.82	339.21
	***f***	0.2334	0.0000	0.0064	0.0031	0.0000	0.0941

**Table 2.** Six lowest triplet excitation energies (*E*, eV) and wavelengths (nm) of chrysophanol.

		**T_1_**	**T_2_**	**T_3_**	**T_4_**	**T_5_**	**T_6_**
**Vacuum**	***E***	2.2354	2.2534	2.5311	2.8199	3.0092	3.3447
	**λ**	554.64	550.22	489.85	439.68	412.02	370.69
**Water**	***E***	2.2128	2.2583	2.6887	2.7439	2.9899	3.3540
	**λ**	560.31	549.02	461.13	451.85	414.68	369.66
**Ether**	***E***	2.2124	2.2491	2.6004	2.7729	2.9986	3.3497
	**λ**	560.39	551.27	476.79	447.13	413.47	370.13

**Table 3.** Total electronic energies of parent molecule (*E*_p_, in hartree), anion radical (*E*_a_, in hartree) and cation radical (*E*_c_, in hartree).

	***E*_p_**	***E*_a_**	***E*_c_**	**VEA_S0_^a^**	**VIP_S0_^b^**
**Vacuum**	−878.5807956	−878.6332437	−878.286792	−1.43	8.00
**Water**	−878.5935825	−878.7031555	−878.3534459	−2.98	6.53
**Ether**	−878.5823983	−878.6829094	−878.3336513	−2.73	6.77

^a^ VEA_S0_ = *E*_a_ – *E*_p_.

^b^ VIP_S0_ = *E*_c_– *E*_p_.

**Table 4.** Lowest triplet excitation energies (*E*_T1_ in eV), Vertical electron affinities ( VEAs in eV ) and vertical ionization potentials ( VIPs in eV ) of chrysophanol in water and ether.

	***E*_T1_**	**VEA_T1_^a^**	**VEA_S0_**	**VIP_T1_^b^**	**VIP_S0_**
**Vacuum**	2.23	−3.66	−1.43	5.76	8.00
**Water**	2.21	−5.19	−2.98	4.32	6.53
**Ether**	2.21	−4.95	−2.73	4.56	6.77

^a^ VEA_T1_ = VEA_S0_ – E_T1_.

^b^ VIP_T1_ = VIP_S0_ – E_T1_.
